# Phenotypic variability of TRPV4 related neuropathies

**DOI:** 10.1016/j.nmd.2015.03.007

**Published:** 2015-06

**Authors:** Teresinha Evangelista, Boglarka Bansagi, Angela Pyle, Helen Griffin, Konstantinos Douroudis, Tuomo Polvikoski, Thalia Antoniadi, Kate Bushby, Volker Straub, Patrick F. Chinnery, Hanns Lochmüller, Rita Horvath

**Affiliations:** a John Walton Muscular Dystrophy Research Centre, MRC Centre for Neuromuscular Diseases, Institute of Genetic Medicine, Newcastle University, Newcastle upon Tyne, UK; b Institute of Neuroscience, Newcastle University, Newcastle upon Tyne, UK; c Bristol Genetic Laboratory, Pathology Sciences, Southmead Hospital, Bristol, UK

**Keywords:** Axonal neuropathy, Skeletal dysplasia, Transient receptor potential vanilloid 4 gene, Hereditary motor and sensory neuropathy type 2C, Scapuloperoneal spinal muscular atrophy, Congenital distal spinal muscular atrophy

## Abstract

Mutations in the transient receptor potential vanilloid 4 (*TRPV4*) gene have been associated with autosomal dominant skeletal dysplasias and peripheral nervous system syndromes (PNSS). PNSS include Charcot–Marie–Tooth disease (CMT) type 2C, congenital spinal muscular atrophy and arthrogryposis and scapuloperoneal spinal muscular atrophy. We report the clinical, electrophysiological and muscle biopsy findings in two unrelated patients with two novel heterozygous missense mutations in the *TRPV4* gene. Whole exome sequencing was carried out on genomic DNA using Illumina Truseq^TM^ 62Mb exome capture. Patient 1 harbours a de novo c.805C > T (p.Arg269Cys) mutation. Clinically, this patient shows signs of both scapuloperoneal spinal muscular atrophy and skeletal dysplasia. Patient 2 harbours a novel c.184G > A (p.Asp62Asn) mutation. While the clinical phenotype is compatible with CMT type 2C with the patient's muscle harbours basophilic inclusions. Mutations in the *TRPV4* gene have a broad phenotypic variability and disease severity and may share a similar pathogenic mechanism with Heat Shock Protein related neuropathies.

## Highlights

• 2 novel heterozygous missense mutations in the *TRPV4* gene.
• Clinical, and muscle biopsy findings in two patients.
• Description of an overlapping syndrome.
• Muscle biopsy with basophilic inclusions.
• Discussion about possible pathogenic mechanisms.

## 1 Introduction

TRPV4 (transient receptor potential vanilloid 4 channel; OMIM 605427) is a calcium permeable non-selective cation channel expressed in several tissues and cell types [1]. In bone it is expressed in osteoblasts, osteoclasts and chondrocytes and may be involved in bone remodelling. In the peripheral nervous system TRPV4 expression was demonstrated in the skin sensory receptors, in the dorsal root ganglia and to a lesser extent in the motor neurons [2]. The precise role of TRPV4 in neurons has not yet been fully elucidated. TRPV4 is also expressed in smooth muscle cells [1] and in mouse skeletal muscle [3].

The *TRPV4* gene (NM_021625.4) is located on chromosome 12q23-q24.1 and is composed of 15 exons coding 5 different splice variants. Only 2 of the splice variants, TRPV4A and TRPV4D, are processed by the endoplasmic reticulum and incorporated into the plasma membrane [1]. The longest isoform (isoform a) of TRPV4 comprises 871 amino acids and has 2 intracellular domains, namely the N- and C-termini, and six transmembrane alpha–helix domains. The N-terminus is composed of six ankyrin repeats and the C-terminus consists of several calmodulin binding sites [4,5].

Dominant mutations in *TRPV4* have been described in both peripheral nervous system and skeletal diseases. PNSS include hereditary motor and sensory neuropathy type 2C or Charcot–Marie–Tooth disease type 2C (HMSN2C or CMT2C; OMIM 606071), congenital spinal muscular atrophy and arthrogryposis (CSMAA; OMIM 600175) and scapuloperoneal spinal muscular atrophy (SPSMA; OMIM 181405). Vocal cord paralysis and sensorineural hearing deficit were frequently associated findings in patients with neuropathies. Among the skeletal dysplasias, *TRPV4* mutations have been described in patients with brachyolmia (OMIM 113500), spondylometaphyseal dysplasia Kozlowski type (SMD-K; OMIM 184252), metatropic dysplasia (OMIM 156530), parastremmatic dysplasia (OMIM 168400), and spondyloepimetaphyseal dysplasia Maroteaux type (SEMD-M; OMIM 184095). There are only a few patients reported with the combination of peripheral neuropathy and skeletal dysplasia [6,7]. A few muscle pathology descriptions in TRPV4-related neuropathies point towards a chronic neurogenic process without any specific or distinguishable features [7].

Here we report two patients, one manifesting a combination of scapuloperoneal spinal muscular atrophy and skeletal dysplasia while the other presented with a CMT2C phenotype and basophilic inclusions in the muscle biopsy.

## 2 Patients and methods

### 2.1 Patients

#### 2.1.1 Patient 1

This 8-year-old boy is the second child of non-consanguineous parents. He was born at 36 weeks gestation by caesarean section due to pre-eclampsia. His birth weight was 2.64 kg and the Apgar score was normal. There is no family history of a neuromuscular condition. His mother is healthy while his father suffers from ankylosing spondylitis. At the age of 10 weeks he was diagnosed with a torticollis and noted to be a floppy baby with difficulties in moving his legs and his left arm. Due to delayed motor development he was able to sit only at the age of 8 months and he walked at the age of 22 months. He had a mild expressive language delay; being unable to pronounce clear words by the aged of 24 months. He was first seen at the age of 30 months due to unsteady gait, inability to jump or run and frequent falls. There were minor swallowing problems both with solids and liquids.

**Fig. 1:**
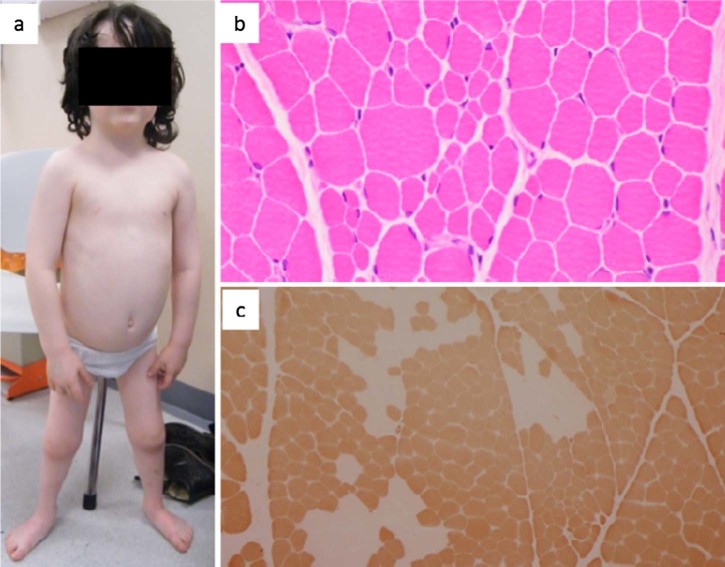
(a) Picture of patient 1 showing the disproportion between the size of the trunk and legs, short stature, short lower limbs, brachydactyly, flat feet and genus valgus. (b) Muscle biopsy (H&E) showing increased variation in fibre size with both scattered and small groups of atrophic fibres. (c) Muscle biopsy (ATPase 4.3) revealing type 1 fibre predominance and a tendency for type-grouping.

There was a slight lumbar lordosis, short stature, short lower limbs, brachydactyly, flat feet and genus valgus, suggestive of skeletal dysplasia. Physical examination (Fig. 1) revealed intact cranial nerves. The eye movements and hearing were normal; there was no tongue fasciculation or facial weakness. Proximal and distal lower limb weakness and proximal upper limb weakness were noted bilaterally. Muscle wasting was present in the lower limbs with a more pronounced effect on the distal muscle groups. There was mild scapular winging, marked waddling gait, bilateral foot drop and a positive partial Gower's manoeuvre. Deep tendon reflexes were absent in the lower limbs. There were no sensory changes, signs of tremor, ataxia, dystonia or joint hyperlaxity.

Laboratory investigations including serum CK level were unremarkable. Spinal cord MRI scan did not show any abnormalities. Motor and sensory nerve conduction velocities of the lower limbs were normal. Needle electromyography revealed a chronic neurogenic pattern with minimal active denervation. Video fluoroscopy showed weak oral stage with poor bolus control. There was no respiratory or cardiac involvement. Genetic analysis did not detect mutations in *SMN1*, *IGHMBP2*, *MFN2* or *FSHD1* genes.

#### 2.1.2 Patient 2

Patient 2 is a 48-year-old female, who had normal motor development and motor function until the age of 40 years. There was no family history of a neuromuscular condition, but her younger brother was born with bilateral talipes. He was unable to attend our clinic for neurological and electrophysiological examinations. The first symptom of patient 2 was recurrent twisting of the right ankle, which occurred by the age of 40 years. There were progressive walking difficulties, due to lower limb weakness. There were no cardiac or respiratory manifestations.

On physical examination cranial nerves were normal. Bilateral foot drop, steppage gait and weakness of the lower limbs were observed. In the proximal segments the strength was grade 4/5 and in the distal ones grade 2/5 according to Medical Research Council Scale for muscle strength. Deep tendon reflexes were normal except for the patella reflexes which were pathologically brisk. The plantar reflex response was flexor. There was minor pinprick and vibration sensory loss in the toes. Muscle tone and bulk were normal.

**Fig. 2:**
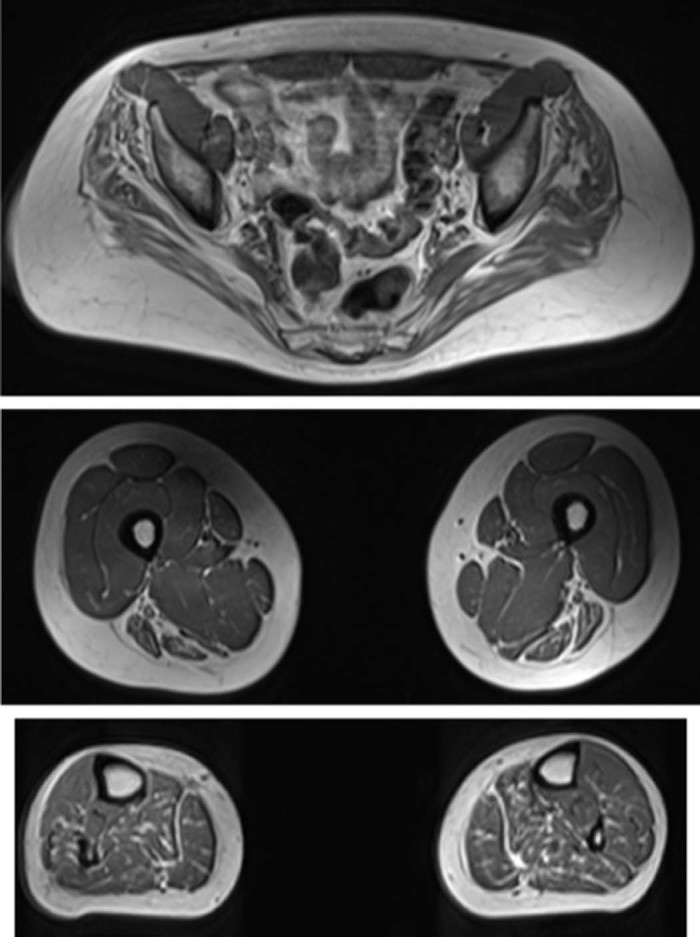
Muscle MRI, T1-weighted axial images, symmetrical atrophy and fat infiltration of the gluteus, hamstrings and calf muscles.

Laboratory tests including serum CK level were unremarkable. Brain and spinal cord MRI scans did not show any lesions. Motor and sensory nerve conduction velocities of the lower limbs were normal, but reduced motor amplitudes in the right peroneal (1.8 mV) and right tibial (5 mV) and absent F-waves were observed. Upper limb motor studies were normal but the amplitude of sensory potential in the right sural nerve was reduced. Needle electromyography revealed minimal active denervation and chronic neurogenic changes with occasional fibrillation potentials. Muscle MRI (Fig. 2) showed, on T1-weighted axial images, symmetrical atrophy and fat infiltration of the gluteus, hamstrings and calf muscles. Diagnostic genetic testing did not identify mutations in *SMN1*, *HSPB1* and *HSPB8* genes.

### 2.2 Methods

#### 2.2.1 Muscle biopsy

Both patients underwent open muscle biopsy from the left quadriceps for patient 1 and left tibialis anterior for patient 2. The biopsies were processed according to standard methods [8].

#### 2.2.2 Genetic analysis

Whole-exome sequencing was carried out in both patients using genomic DNA extracted from peripheral blood lymphocytes, fragmented, and exome enriched by Illumina TruSeq™ 62 Mb and sequenced on a HiSeq 2000 with 100 bp paired-end reads. Bioinformatic analysis was performed using an in-house algorithm based on published tools. The sequence was aligned to the human reference genome (UCSC hg19) using Burrows–Wheeler Aligner and reformatted using SAMtools. Duplicated sequence reads were removed (Picard v.185) and variants were identified using VarScan (v.2.2) and Dindel (v1.01).

Results were further filtered for variants with a minor allele frequency less than 0.01 in several databases: dbSNP135, 1000 genomes (February 2012 data release), the National Heart, Lung and Blood Institute (NHLBI, NIH, Bethesda, MD) Exome Sequencing Project (ESP) 6500 exomes, and 238 unrelated in-house controls, in order to detect rare variants. We selected candidate variants among known neuropathy-related disease genes [9].

Patient 2 was tested in parallel for a panel of 56 genes associated with inherited peripheral neuropathies, using Agilent SureSelectXT^2^ custom target enrichment system and Next Generation Sequencing. We carried out PCR (IMMOLASE™ DNA Polymerase, Bioline UK) and Sanger sequencing (BigDye® Terminator v3.1) of variants which were predicted to be deleterious by three online prediction tools (MutationTaster, SIFT and Polyphen2).

## 3 Results

### 3.1 Muscle histology

In patient 1 (Fig. 1) muscle biopsy showed increased variation in fibre size with both scattered and small groups of atrophic fibres. ATPase stain showed type I fibre predominance with many of the fascicules being entirely type I. In some fascicules there were areas of fibre type grouping, indicative of a chronic neuropathy.

**Fig. 3:**
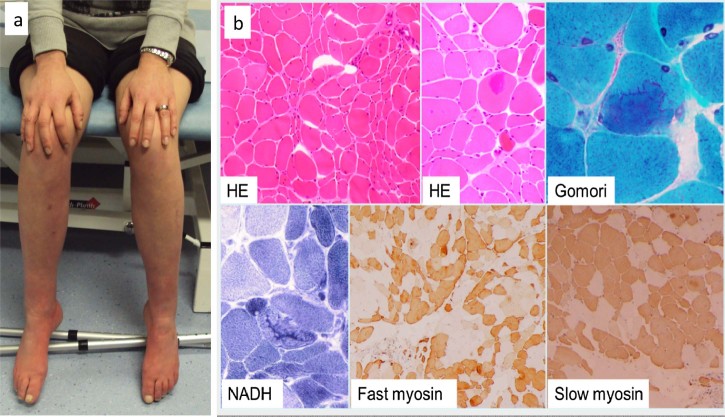
(a) Picture of patient 2 shows the severe weakness in the feet. (b) Muscle biopsy from patient 2 showing neurogenic atrophy and large accumulations of basophilic material.

In patient 2 (Fig. 3) there was evidence of neurogenic atrophy, increased muscle fibre size variation with both hypertrophic and atrophic fibres. The atrophic fibres occurred in clusters and some were angulated. There was a tendency to fibre type grouping and an increase in the number of internal nuclei. Large, mainly central accumulation of basophilic material was observed in occasional fibres (Fig. 3).

### 3.2 Genetic analysis

Patient 1 had a de novo, heterozygous missense mutation c.805C > T (p.Arg269Cys) in exon 5 of the *TRPV4* gene. This mutation has been repeatedly reported in previous studies [10]. Both parents tested negative for this mutation. Patient 2 had a novel heterozygous variant c.184G > A (p.Asp62Asn) in exon 2 of the *TRPV4* gene. This novel variant was absent in the healthy mother and was present in her brother, the father was deceased and DNA was not available for testing. The sequence change is predicted to cause the substitution of a moderately conserved aspartic acid for an arginine at codon 62 in the N-terminal cytoplasmic domain of the protein that contains six ankyrin repeats. The theoretical prediction (SIFT, Polyphen 2 HDIV, Polyphen 2 HVar, Mutation taster) based on *in silico* analysis suggests that this variant may have a deleterious effect on the protein function. This variant was not reported in dbSNP or the ESP database, it has been seen once in 119,558 alleles (http://exac.broadinstitute.org/variant/12-110252418-C-T), and this rarity supports pathogenicity. No other potentially deleterious variant was detected in any known myopathy or neuropathy genes on whole exome sequencing.

## 4 Discussion

Here we report two patients with different clinical presentations carrying pathogenic mutations in the *TRPV4* gene. Patient 1 has a phenotype combining scapuloperoneal spinal muscular atrophy (SPSMA) and metatropic dysplasia. A similar combination was reported previously in 3 patients [6] with either spondylometaphyseal dysplasia Kozlowski type or spondyloepimetaphyseal dysplasia Maroteaux type and HSMN type II axonal neuropathy. Our patient has a clear clinical and neurophysiological phenotype of SPSMA, supporting the existence of combined skeletal muscle and nerve involvement. Minor skeletal abnormalities in association with a neuropathy were reported in some patients with *TRPV4* mutations previously [7,11–13]. The mutation c.805C > T (p.Arg269Cys) was described in patients with HSMN type IIC, with SPSMA and also with distal congenital non progressive SMA [7,12,14–16], suggesting significant clinical heterogeneity. To our knowledge this mutation has never been associated with skeletal dysplasia; however milder skeletal abnormalities may have been missed on routine neurological examination. Our finding expands further the clinical variability among *TRPV4* mutations and highlight that the presence of skeletal abnormalities in a patient with an axonal neuropathy or neuronopathy should raise the possibility of *TRPV4* gene mutations.

Patient 2 presents with a phenotype compatible with CMT2C. Although the mutation c.184G > A (p.Asp62Asn) has not been previously reported, it was also present in the patient's 29 year old brother who had congenital bilateral talipes, but it was absent in the asymptomatic mother. The *in silico* studies and mutation prediction programmes indicate that this variant is probably pathogenic by changing the conformation of the protein.

There are only a few reports on muscle histology in patients with *TRPV4* mutations. In most cases muscle pathology demonstrated abnormalities consistent with chronic denervation [7,17], such as in patient 1. These included increased variation in fibre size with hypertrophic and scattered or small clusters of atrophic muscle fibres and fibre type grouping, which could be explained by the neuropathy. Interestingly patient 2 presented with intra-cytoplasmic basophilic inclusions besides the neurogenic changes. *In vitro* studies in HeLa cells showed that mutated TRPV4 protein forms cytoplasmic aggregates without localizing to the plasma membrane where wild-type TRPV4 proteins are usually detected [10].

The accumulation and aggregation of misfolded proteins can be highly cytotoxic and can lead to several neurodegenerative conditions. Misfolded proteins tend to aggregate and precipitate in the cells triggering different cytotoxic pathways. It is hypothesized that aggregation of misfolded proteins and formation of cytoplasmic aggregates are the main disease pathways in neuropathies associated with mutations in the heat shock protein genes (HSP) [18,19]. We speculate that *TRPV4* mutations are associated with the production of misfolded proteins, which can form cytoplasmic aggregates similar to HSP related neuropathies. In support of this we emphasize the fact that the muscle biopsy from patient 2 showed intracellular aggregates, but the exact protein composition of these aggregates has not been elucidated yet.

Muscle MRI of the thighs and calf muscles from patients with SMA associated with *TRPV4* mutations was reported as having extensive fat atrophy with the preservation of the biceps femoris and medial gastrocnemius [20]. In another study in patients with *TRPV4* mutations the adductors and semitendinosus muscles were well preserved whereas the quadriceps, vastus medialis, long head of biceps and semimembranosus were abnormal. In the calves, the flexor hallucis longus and peroneus brevis muscles were spared and the gastrocnemius presented with a feather like atrophy [17]. In patient 2 MRI showed symmetrical fat atrophy of the biceps femoris, semitendinosus, semimembranosus and calf muscles. At present, the reports on muscle MRI images of patients with *TRPV4* mutations do not show a characteristic pattern of muscle involvement, most likely due to highly variable clinical presentations. The detected changes are not typical for a peripheral neuropathy affecting mainly distal groups. We assume that the differences between our patient and the ones reported by Astrea et al. [20] and Oates et al. [17] are due to the fact that our case is a late onset form of the disease (CMT2C) while the later ones corresponded to congenital forms of SMA. Therefore muscle MRI is of limited benefit as a diagnostic tool in TRPV4-related diseases.

## 5 Conclusions

In summary, mutations in the *TRPV4* gene lead to a broad spectrum of phenotypic manifestations with marked variability in disease severity. The association with skeletal deformities, in particular short stature, brachydactyly, disproportion between the lower and the upper halves of the body, may help with the differential diagnosis. Vocal cord paralysis and, to a lesser extent, neurosensory deafness are described as clinical clues to diagnose TRPV4 associated neuropathies; however none of these were present in our patients. The presence of intra-cytoplasmic basophilic inclusions in muscle biopsies of patients with axonal neuropathies should raise the possibility of mutations either in *TRPV4* or in heat shock protein genes.
